# Genetic interaction of GSH metabolic pathway genes in cystic fibrosis

**DOI:** 10.1186/1471-2350-14-60

**Published:** 2013-06-10

**Authors:** Fernando Augusto de Lima Marson, Carmen Sílvia Bertuzzo, Rodrigo Secolin, Antônio Fernando Ribeiro, José Dirceu Ribeiro

**Affiliations:** 1 Department of Pediatrics, Faculdade de Ciências Médicas, Universidade Estadual de Campinas - Unicamp, 13081-970, P.O. Box: 6111, Campinas, SP, Brazil; 2 Department of Genetics, Faculdade de Ciências Médicas, Universidade Estadual de Campinas - Unicamp, 13081-970, P.O. Box: 6111, Campinas, SP, Brazil

## Abstract

**Background:**

Cystic fibrosis (CF) is a monogenic disease caused by *CFTR* gene mutations, with clinical expression similar to complex disease, influenced by genetic and environmental factors. Among the possible modifier genes, those associated to metabolic pathways of glutathione (GSH) have been considered as potential modulators of CF clinical severity. In this way it is of pivotal importance investigate gene polymorphisms at Glutamate-Cysteine Ligase, Catalytic Subunit (*GCLC*), Glutathione S-transferase Mu 1 (*GSTM1*), Glutathione S-transferase Theta 1 (*GSTT1*), and Glutathione S-transferase P1 (*GSTP1*), which have been associated to the GSH metabolic pathway and CF clinical severity.

**Method:**

A total of 180 CF’s patients were included in this study, which investigated polymorphisms in *GCLC* and *GST* genes (GCLC -129C>T and -3506A>G; *GSTM1* and *GSTT1* genes deletion, and GSTP1*+313A>G) by PCR and PCR-RFLP associating to clinical variables of CF severity, including variables of sex, clinical scores [Shwachman-Kulczycki, Kanga e Bhalla (BS)], body mass index, patient age, age for diagnosis, first clinical symptoms, first colonization by *Pseudomonas aeruginosa*, sputum’s microorganisms, hemoglobin oxygen saturation in the blood, spirometry and comorbidities. The *CFTR* genotype was investigated in all patients, and the genetic interaction was performed using MDR2.0 and MDRPT0.4.7 software.

**Results:**

The analysis of multiple genes in metabolic pathways in diseases with variable clinical expression, as CF disease, enables understanding of phenotypic diversity. Our data show evidence of interaction between the *GSTM1* and *GSTT1* genes deletion, and GSTP1*+313A>G polymorphism with *CFTR* gene mutation classes, and BS (Balance testing accuracy= 0.6824, p= 0.008), which measures the commitment of bronchopulmonary segments by tomography.

**Conclusion:**

Polymorphisms in genes associated with metabolism of GSH act on the CF’s severity.

## Background

Cystic Fibrosis (CF) is a common disease in Caucasian population, with a prevalence of 1:2,500 live births [1]. CF shows an autosomal recessive pattern of inheritance [2], which is caused by mutations in Cystic Fibrosis Transmembrane Regulator (*CFTR*) gene. More than 1,900 mutations in *CFTR* gene have been associated to CF clinical severity [3-6]. However, in the pulmonary disease, which is the main cause of CF morbidity and mortality, several studies have demonstrated that clinical variability is influenced by modifier genes and environmental factors [2,7].

Most of modifier genes related to CF have been associated with chloride transportation, infection and inflammation in the lungs [2,7-10]. In our group, as previously published, multiple genes are associated with CF clinical severity, including Transforming growth factor beta 1 (*TGF*-*β1*) [11], Glutathione S-transferase Mu 1 (*GSTM1*), Glutathione S-transferase Theta 1 (*GSTT1*) [12], Angiotensin-converting enzyme (*ACE*) [13] and Beta-2-Adrenergic Receptor (*ADBR2*) [14] genes as possibly modifier genes. In the same population the mannose-binding lectin (protein C) 2 (*MBL2*) and monocyte differentiation antigen CD14 (*CD14*) genes were not associated with the CF clinical severity [11].

Glutathione (GSH) is a tripeptide involved in the intracellular defense system, which protect the epithelium against injuries and inflammation, both common events of CF [15]. Polymorphisms in regulator genes of GSH metabolic pathway have been described in CF, including Glutathione S-transferase P1 (*GSTP1*), *GSTT1*, *GSTM1* and Glutamate-Cysteine Ligase, Catalytic Subunit (*GCLC*) genes, which have been associated with greater CF clinical severity [7,12,16-23]. In the other hand, *GCLC* [129C>T and -3506A>G] polymorphisms gene has not been yet evaluated for pulmonary disease variability in CF.

*GCLC* gene encodes the catalytic subunit of glutamate-cysteine ligase (GCL) [24], which is the first limiting enzyme in the GSH synthesis [25]. Polymorphisms -129C>T and -3506A>G, located in *GCLC* gene promoter region, have been associated to reduced GSH production [15,25].

The Glutathione S-transferase (GST) is a family of enzymes, which associates GSH and causative compounds of oxidative stress, to a wide variety of endogenous (e.g. by-products of reactive oxygen species activity) and exogenous (e.g. polycyclic aromatic hydrocarbons) electrophilic substrates [26]. Polymorphisms in *GST* family genes can also be involved in CF’s severity [15,26], especially in pulmonary disease, including *GSTM1*, *GSTT1* and *GSTP1* genes [15,19,20,26-28].

In our referral center, we have observed clinical variability among CF’s patients. However, the patients present similar socioeconomic status, none severe malnutrition, mutations of classes I, II and/or III, similar support of the Parents Association (http://www.fibrocis.org.br), and receive free medication by the government. Therefore, we postulate that clinical variability could be associated with interaction of polymorphisms in modifier genes. In this context, we aim to analyze polymorphisms in *GCLC* and *GST* genes (*GCLC* -129C>T and -3506A>G; *GSTM1* and *GSTT1* genes deletion, and GSTP1*+313A>G) associating to clinical markers of CF severity.

## Methods

### Ascertainment of patients

It was conducted a cross-sectional study and patients were selected during the period of 2010 and 2011, in a university center for CF patients care. This study was approved by Ethics Research Board of the Faculty of Medical Sciences at University of Campinas - São Paulo – Brazil (#528/2008). The study was in accordance to the Helsinki declaration and all patients signed a consent form before beginning the study. For patients below the age of 18 the consent was granted by their parents or guardians.

Diagnosis of CF was confirmed in patients with two doses of sodium and chloride from the sweat with values greater than 60 mEq/L. In a group of patients we identified two *CFTR* gene mutations. No patient had received neonatal screening test performed for CF. A total of 215 patients were selected for the study. Among them, 35 patients without clinical data for statistical analysis and those who did not sign the consent form were excluded from the study, and 180 CF patients were included at the study.

### Clinical variables

The following clinical variables were employed: clinical scores [Shwachman-Kulczycki (two groups: ≤ 65 and > 65), Kanga (two groups: ≤ 17 and > 17) and Bhalla (BS) (two groups: ≤ 8 and > 8)] [29]; body mass index (BMI) [for the patients older than 19 years the BMI= weight/(height)^2^ formula was used; for the remaining patients the WHO ANTHRO programs (children under 5 years old) and WHO ANTHRO PLUS (children 5 - under 19 years old) [30,31] were used; patient age (group: ≤ 154 and > 154 months) and age at diagnosis (according to the sodium and chloride in altered perspiration: ≤ 25 and > 25 months); first clinical symptoms [(digestive: ≤ 4 and > 4 months; pulmonary: ≤ 7 and > 7months)]; the period up to first colonization by *Pseudomonas aeruginosa* (≤ 31 and > 31 months); sputum’s microorganisms [*P*. *aeruginosa* mucoid and non mucoid, *Achromobacter xylosoxidans*, *Burkolderia cepacia* and *Staphylococcus aureus*]; hemoglobin oxygen saturation in the blood (≤ 96 and > 96); spirometry and comorbidities: nasal polyps, osteoporosis, meconium ileus, diabetes mellitus and pancreatic insufficiency (PI).

The clinical score evaluation was performed by two specialists in double-blind analyses, and in discordance case, another one was consulted. The Shwachman-Kulczycki score compares clinical manifestations among patients, detect treatment effects and aid in the determination of diagnostic criteria. To that end, the system evaluates 4 major parameters: general activity, nutrition, chest radiographic findings and physical examination. The score for each parameter ranges from 5 to 25. Lower score’s values are associated with CF severity [29]. The BS is a tomographic scoring system to assess pulmonary involvement, determine therapeutic effects and aid selection of patients for transplants, with small variation between the scores given by the various examiners and the score proved to have good reproducibility and high correlation with pulmonary function test results. A total of 9 categories, worth 3 points each, are scored, and a maximal score equals a high degree of severity. The final score must be subtracted from 25. Lower score’s values are associated with CF severity [29]. Kanga score is a system designed to assess acute exacerbations of the disease, to predict improvement or worsening of pulmonary function and to evaluate therapeutic effects with little variability between examiners and correlated significantly with pulmonary function (FEV_1_ and forced vital capacity) test results. The system helps identify daily clinical changes and includes 5 common symptoms (cough, fluid secretion, appetite loss, dyspnea and frailty) and 5 physical signs (temperature, weight, respiratory frequency, wheezing and respiratory sounds). Each criterion is worth 1 to 5 points. Higher score’s values are associated with CF severity [29].

Spirometry was performed in patients older than 7 years, using the CPFS/D spirometer (MedGraphics, Saint Paul, Minnesota, USA). Data was recorded by the PF BREEZE software version 3.8B for Windows 95/98/NT [32] and the following markers were included: forced vital capacity [FVC(%) - ≤ 82 and > 82], forced expiratory volume in the first second [FEV_1_(%) - ≤ 72 and > 72]_,_ FEV_1_/FVC(%) - ≤ 86 and > 86] and forced expiratory flow between 25 and 75% of the FVC [FEF_25-75_% - ≤ 57 and > 57].

### DNA extraction and polymorphisms genotyping

DNA sample was obtained from peripheral blood using phenol-chloroform standard procedure. In order to evaluate DNA concentration, we quantified the entire sample using GE NanoVue™ Spectrophotometer (GE Healthcare Biosciences, Pittsburgh, USA).

The *CFTR* gene mutations were investigated by PCR technique (F508del) and the restriction fragment length polymorphism (RFLP) method (G542X, R1162X, R553X, G551D and N1303K). Some mutations in CF patients were obtained by sequencing or MLPA (Multiplex Ligation - dependent Probe Amplification) analysis: S4X, 2183A>G, 1717-G>A and I618T. For sequencing and MLPA, we used the same MegaBace1000® (GE Healthcare Biosciences, Pittsburgh, USA) [33]. The *CFTR* genotype separated the patients in 3 groups: (1) patients with two mutations identified classes I, II and/or III, (2) patients with one mutations identified classes I, II or III, (3) patients with no identified mutation. All mutations identified were included in the class one, two or three of the *CFTR* gene. Other identified mutations, class IV (P205S e R334W) were not included in the statistical analysis.

For the *GSTM1* and *GSTT1* genes deletion, a multiplex PCR reaction was performed and the *CYP1A1* gene was included as an internal reaction control [34]. In the assay tests for the wild type allele, the samples for which no signal is obtained are homozygous for the deletion. Heterozygotes and homozygotes for the wild-type allele cannot be differentiated by the multiplex technique used. As *GSTM1* and *GSTT1* deletion analyze not provide complete genotypes, minor allele frequencies and Hardy-Weinberg Equilibrium cannot be evaluated for these loci in our study. By the method realized we have two groups of *GSTM1* and *GSTT1* gene: (i) patients homozygous to gene deletion; (2) patients with at least an allele expressed. The RFLP was performed for polymorphisms GCLC*-129C>T, GCLC*-3506A>G [10,15] and GSTP1*+313A>G [28].

Other genes were studied in the same CF reference center with same population as previously published [11,13,14]. The *GSTM1* and *GSTT1* genes were studied in our center considering other CF patients as recent published data showed [12]. The patient’s studied by Lima et al. (2012) [12] did not include the same CF patients, and in the previously study, only the follow clinical variables were employed: age, sex, ethnic and Shwachman-Kulczycki.

### Statistical analysis

To develop statistical analyses, patients were divided into two subgroups according to clinical variables distribution. We evaluate minor allele frequency (MAF) and Hardy-Weinberg equilibrium (HWE) using OEGE software (Online Encyclopedia for Genetic Epidemiology studies, 2008). In order to evaluate genetic interaction among the polymorphisms in our sample, we used the Multifactor Dimensionality Reduction (MDR) model, which is a nonparametric and genetic model-free data mining for nonlinear interaction identification among genetic and environmental attributes [35-37]. To adjust results for multiple comparisons, we performed a MDR permutation test in our sample, totalizing 100,000 permutations.

## Results and discussion

The CF is a disease with a high phenotypic variability, resulting from the interaction between genetic and environmental factors, which could affect differential expression between patients with similar *CFTR* gene mutations [2]. Despite CF’s severity is difficult to characterize, we describe 28 clinical variables (Table 1), consistent with the phenotype feature of the disease.

**Table 1:** Clinical features of cystic fibrosis patients included in the study

	
Sex (male)	50% (80)^#^
Age	17.72 ± 15.75 years (0.6 – 24 years)*
BMI - thinness and accentuated thinness	22.22% (40)^#^
One Class I. II or III identified mutation	28.33% (51)^#^
Two Class I. II or III identified mutation	47.22% (85)^#^
First clinical manifestation	2.90 ± 8.89 years (0 – 13 years)*
Age at diagnosis	7.62 ± 13.63 years (0 – 14.23 years)*
Onset of digestive symptoms	3.39 ± 9.11 years (0 – 12.45 years)*
Onset of pulmonary symptoms	2.90 ± 9.89 years (0 – 13 years)*
SpO2	94.92 ± 4.26 (66 – 99)*
Bhalla score	8.74 ± 5.724 (0 – 25)*
Kanga score	18.85 ± 5.84 (10 – 40)*
Shwachman-Kulczycki score	65.85 ± 16.77 (20 – 95)*
FVC (%)	79.29 ± 23.55 (19 – 135)*
FEV_1_ (%)	71.29 ± 27.47 (17 – 132)*
FEV_1_/FVC (%)	83.46 ± 15.95 (37 – 137)*
FEF_25-75_%	59.05 ± 35.55 (7 – 150)*
Nasal Polyps	18.33% (33)^#^
Diabetes mellitus	18.33% (33)^#^
Osteoporosis	16.11% (29)^#^
Pancreatic insufficiency	80.0% (144)^#^
Meconium ileus	15.00% (27)^#^
First isolated *P*. *aeruginosa*	8.55 ± 14.45 years (2 – 15 years)
*P*. *aeruginosa* status	56.67% (102)^#^
*P*. *aeruginosa* mucoid status	42.22% (76)^#^
*B*. *cepacia* status	13.88% (25)^#^
*A*. *xylosoxidans* status	10.00% (18)^#^
*S*. *aureus* status	78.88% (142)^#^

*BMI* body mass index, *SpO2* Hemoglobin oxygen saturation in the blood, % percentage, *FVC* forced vital capacity, *FEV*_*1*_ forced expiratory volume in the first second, *FEF*_*25*-*75*_ forced expiratory flow between 25 and 75% of FVC. 2. Based on 3 Consecutive positive respiratory cultures.

# Percentage (Number of patients).

* Continuous variables expressed as mean ± SD (range).

Analyses of *GCLC*, *GSTM1*, *GSTT1*, and *GSTP1* genes polymorphism is shown in Table 2. The *CFTR* mutation showed two different mutations in 85 (47.22%) CF patients; only one mutation in 51 (28.33%) CF patients and none mutation in 44 (24.44%) CF patients (Table 2).

**Table 2:** **Genotypic characteristic of gene polymorphisms at *GCLC***, ***GSTM1***, ***GSTT1***, **and *GSTP1 *genes and *CFTR *gene mutation among cystic fibrosis patients**

**Gene**	**Chromosome position**	**Location**	**Variation**	**Genotype**			**MAF**	p*
				C/C	C/T	T/T		
*GCLC*, rs17883901	6p12	Promoter region	C/T	144 (80%)	29 (16.11%)	7 (3.89%)	0.12	<0.005 ^1^
				A/A	A/G	G/G		
*GCLC*, rs137852340	6p12	Promoter region	A/G	118 (65.56%)	56 (31.11%)	6 (3.33%)	0.19	>0.05
*GSTP1*, rs1695	11q13	Exon 5	A/G	97 (53.89%)	74 (41.11%)	9 (5%)	0.26	>0.05
				Wt/Wt + Wt/del		del/del		
*GSTM1*	1p13.3		Deletion	108 (60%)		72 (40%)		
*GSTT1*	22q11.23		Deletion	117 (65%)		63(35%)		
***CFTR *mutation genoytpe**		**N**		**Frequency**				
F508del/F508del		57		31.67%				
F508del/G542X		12		6.67%				
F508del/R1162X		5		2.78%				
F508del/N1303K		4		2.22%				
F508del/R553X		1		0.56%				
F508del/S4X		1		0.56%				
F508del/1717-1G>A		1		0.56%				
G542X/R1162X		1		0.56%				
G542X/I618T		1		0.56%				
G542X/2183A>G		1		0.56%				
R1162X/R1162X		1		0.56%				
F508del/-		45		25.00%				
G542X/-		5		2.78%				
R1162X/-		1		0.56%				
−/−		44		24.45%				

*GCLC* glutamate-cysteine ligase catalytic subunit, *GSTM1* Glutathione S-transferase Mu 1, *GSTT1* Glutathione S-transferase theta 1, *GSTP1* Glutathione S-transferase P1, *CFTR* Cystic fibrosis transmembrane conductance regulator, *C* Cytosine, *T* Thymine, *A* Adenine, *G* Guanine, < minor than, > bigger than, *MAF* minor allele frequency, % percentage, *p value for Hardy-Weinberg Equilibrium, *N* number of patients, *Wt* Wild allele, *del* deleted allele, (−) *CFTR* mutation no identified. 1- *GCLC*, rs17883901 is not in Hardy-Weinberg Equilibrium in our sample.

The polymorphism GCLC-129C>T is not in Hardy-Weinberg equilibrium. The MAF observed in present data is 0.012, and the data achieved at National Center for Biotechnology Information (http://www.ncbi.nlm.nih.gov/), by HapMap project, shows a MAF of 0.076. The observed difference can be a strong factor to consider the polymorphism as an important modifier of CF severity, but we did not find in our data genotype-phenotype-association. Maybe, with a bigger population and other clinical variables can be possible to determine the polymorphism influence. Another factor should be considered that is the Brazilian population is an admixture population, what can provide changes in the clinical associations.

The MDR analysis showed evidence of interaction of *GSTM1* and *GSTT1* genes deletion, GSTP1*+313A>G, and *CFTR* mutations (p= 0.008) and BS clinical score (Table 3). All data was previously associated with a point-point analysis considering which gene polymorphism in association with one clinical manifestation at a time by *CFTR* groups, and after Bonferroni correction, we did not observed positives p-value (Additional file 1: Table S4, Additional file 2: Table S5, Additional file 3: Table S6, Additional file 4: Table S7, Additional file 5: Table S8). In this context, the data by gene-gene interaction provides a better tool to contribute in the clinical manifestation association. The association observed cannot identify if those patients who carry risk alleles at all four loci will have the most severe clinical phenotype reflected by the BS.

**Table 3:** **Genetic interaction of polymorphisms and *CFTR *mutations in association with cystic fibrosis clinical variables**

**Clinical variables**		**Gene**	**Ratio**	**Testing ball. acc.**	**p-value**	**Cross validation consistency**
Male/female		CFTR*GCLC*GSTP1*GSTT1*GSTM1	1	0.5056	0.8506	10/10
Race (caucasian/ no caucasian)		CFTR*GCLC*GSTP1*GSTT1*GSTM1	0.0909	0.3909	0.9915	10/10
Age		CFTR*GCLC*GSTP1*GSTT1*GSTM1	0.9889	0.5312	0.6870	10/10
Age for diagnosis		CFTR*GCLC*GSTP1*GSTT1*GSTM1	0.8804	0.4100	0.9989	10/10
Onset of symptoms	geral	CFTR*GCLC*GSTP1*GSTT1*GSTM1	0.7526	0.4286	0.9945	10/10
	digestive	CFTR*GCLC*GSTP1*GSTT1*GSTM1	0.9737	0.4614	0.9698	10/10
	pulmonary	CFTR*GCLC*GSTP1*GSTT1*GSTM1	0.7935	0.5022	0.8566	10/10
Clinical scores	Bhalla	CFTR*GCLC*GSTP1*GSTT1*GSTM1	0.9552	0.6824	**0**.**0080**^#^	10/10
	Kanga	CFTR*GCLC*GSTP1*GSTT1*GSTM1	0.9571	0.5131	0.8165	10/10
	Shwachman-Kulczycki	CFTR*GCLC*GSTP1*GSTT1*GSTM1	0.9	0.5562	0.5474	10/10
Body mass index		CFTR*GCLC*GSTP1*GSTT1*GSTM1	3.45	0.5484	0.6260	10/10
Sputum’s microbiology	*A. xylosoxidans*	CFTR*GCLC*GSTP1*GSTT1*GSTM1	0.1118	0.4617	0.9418	10/10
	*P*. *aeruginosa* mucoid	CFTR*GCLC*GSTP1*GSTT1*GSTM1	0.7379	0.5505	0.5507	10/10
	*P*. *aeruginosa* no mucoid	CFTR*GCLC*GSTP1*GSTT1*GSTM1	1.2949	0.4151	0.9951	10/10
	*S*. *aureus*	CFTR*GCLC*GSTP1*GSTT1*GSTM1	3.7105	0.5094	0.8317	6/10
	*B*. *cepacia*	CFTR*GCLC*GSTP1*GSTT1*GSTM1	0.1548	0.5277	0.7610	10/10
First isolated *P*. *aeruginosa*		CFTR*GCLC*GSTP1*GSTT1*GSTM1	0.9552	0.4913	0.8903	7/10
Hemoglobin oxygen saturation in the blood		CFTR*GCLC*GSTP1*GSTT1*GSTM1	0.9602	0.5312	0.6904	10/10
Spirometry	FEV_1_	CFTR*GCLC*GSTP1*GSTT1*GSTM1	0.9848	0.4746	0.9448	10/10
	FVC(%)	CFTR*GCLC*GSTP1*GSTT1*GSTM1	0.900	0.4437	0.9833	10/10
	FEV_1_/FVC(%)	CFTR*GCLC*GSTP1*GSTT1*GSTM1	0.9403	0.6426	0.0586	10/10
	FEF_25-75_%	CFTR*GCLC*GSTP1*GSTT1*GSTM1	0.9848	0.5054	0.8447	10/10
Comorbidities	Pancreatic insufficiency	CFTR*GCLC*GSTP1*GSTT1*GSTM1	3.977	0.5266	0.7486	10/10
	Meconium ileus	CFTR*GCLC*GSTP1*GSTT1*GSTM1	0.1776	0.5401	0.6950	10/10
	Nasal polyps	CFTR*GCLC*GSTP1*GSTT1*GSTM1	0.2292	0.5713	0.5139	10/10
	Diabetes mellitus	CFTR*GCLC*GSTP1*GSTT1*GSTM1	0.2292	0.4527	0.9728	10/10
	Osteoporosis	CFTR*GCLC*GSTP1*GSTT1*GSTM1	0.1959	0.3815	0.9988	10/10

*GCLC* glutamate-cysteine ligase catalytic subunit, *GST* glutathione *S*-transferase, Testing Ball. Acc. Testing Balance Accuracy, *CFTRCystic Fibrosis Transmembrane Regulator*, *A*. *xylosoxidansAchromobacter xylosoxidans*, *B*. *cepaciaBurkolderia cepacia*, *P*. *aeruginosaPseudomonas aeruginosa*, *S*. *aureusStaplylococcus aures*, *FEV*_1_ forced expiratory volume in one second, *FVC* - forced vital capacity, *FEF*_25-75_% forced expiratory flow between 25 and 75% of CVF. 1. Severe variants to polymorphism: allele T, *GCLC* 129C>T polymorphism, allele G - GCLC-350A>G and GSTP1+313A>G polymorphisms, deletion allele *GSTM1* and *GSTT1* genes. # Association of GCLC-129C>T, GCLC-350A>G, GSTP1+313A>G, *GSTM1* and *GSTT1* genes deletion and mutations in the *CFTR* gene with the Bhalla Score, **p**: **0**.**0080**. Statistical analysis performed by the MDR2.0 and MDRPT0.4.7 software.

**Figure 1:**
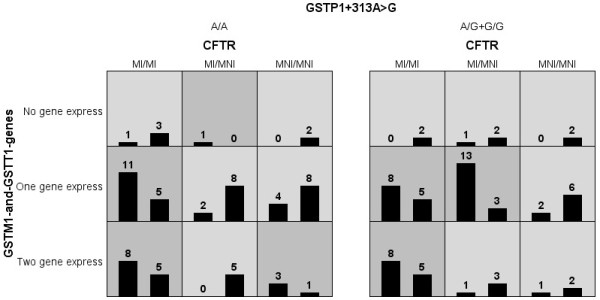
Distribution of patients according to different genotype combinations for the clustering of mutations in the *CFTR *gene and GCLC*-129C>T, GCLC*-350A>G, GSTP1*+313A>G, *GSTM1 *and *GSTT1 *genes deletion polymorphisms with the Bhalla score (BS). For the *CFTR* gene: (MI) Mutation-Identified and (MNI) Mutation-No-Identified to class I, II and/or III. Combinations of high risk are in gray and white to low-risk. In the figure only the polymorphisms with positive interaction with the BS are shown. The stronger association with the BS is observed by the genes with bigger interaction (*GSTM1*, *GSTT1* and *CFTR*), and with the GSTP1*+313A>G polymorphism in a weak association. To data showed, the GCLC*-129C>T, GCLC*-350A>G polymorphisms not show association, and are not included in the figure. *GST* - glutathione *S*-transferase; *CFTR* - Cystic Fibrosis Transmembrane Regulator. The number in the figure represents the patients with genotype combination, for example, in the first square one patient (left column – severe BS) has the follow genotype: (i) two identified mutation in the *CFTR* gene, (ii) deletion of *GSTM1* and *GSTT1* in homozygous, and (iii) A/A genotype to GSTP1+313A>G polymorphism and three (right column- mild BS) patients without the combined genotype presence.

**Figure 2:**
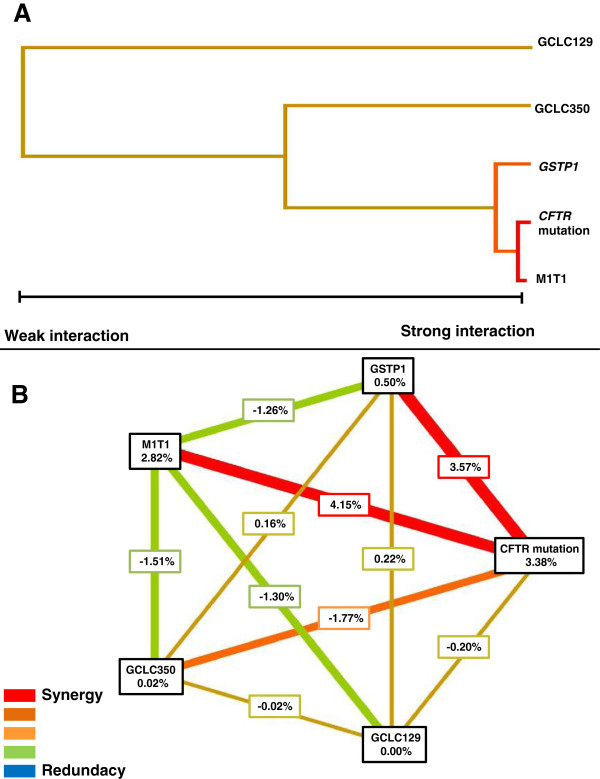
**Dendrogram and graph of entropy with the genes interaction and their polymorphisms in response to Bhalla score. A**. Dendrogram with the genes interaction and their polymorphisms in response to Bhalla score (BS). The stronger interaction of response to Bhalla clinical score is given by *CFTR* mutations and *GSTM1* and *GSTT1* genes deletion. Secondly, the polymorphism GSTP1*+313A> G was associated with the clinical variable. Polymorphisms GCLC*-129C>T and GCLC*-350A>G were not associated with the BS. **B**. Graph of entropy measuring the power of different genotypes and the interaction between them, for the genes analyzed in the gene-gene interaction with BS. The *CFTR* mutation gene, GSTP1*+313A>G and *GSTM1* and *GSTT1* deletion polymorphisms showed significant association with BS. The *CFTR* mutation showed influence of 3.38% for BS, the *GSTM1* and *GSTT1* deletion together, showed 2.82% of influence, while the GSTP1*+313A>G showed influence of 0.5%. The *CFTR* mutation showed a value of 4.15% for the interaction between the deletion polymorphism with *GSTM1* and *GSTT1* genes and 3.57% for the association with GSTP1*+313A>G. The dendrogram construction was performed by the software MDR2.0. The analysis included 180 Cystic Fibrosis patients. # Association of GCLC-129C>T, GCLC-350A>G, GSTP1+313A>G, *GSTM1* and *GSTT1* genes deletion and *CFTR* mutations with the BS show a p-value of 0.0080. *CFTR* - Cystic Fibrosis Transmembrane Regulator; *GCLC* - glutamate-cysteine ligase, catalytic subunit; *GSTP1* - glutathione *S*-transferase P1; *GSTM1* - glutathione *S*-transferase M1; *GSTT1* - glutathione *S*-transferase T1. The power of interaction is show by the distance between the two genes on the figure **A** in horizontal direction. The redundancy/synergy power is show in the figure **B** by the color intensity.

Genotype combinations of the genes that show association with BS clinical score are demonstrated in Figure 1. The largest association regarding to BS clinical response was given by the interaction of *GSTM1* and *GSTT1* genes deletion, and *CFTR* mutations. The polymorphism GSTP1*+313A>G presented entropy with BS clinical score, but with minor intensity (Figure 2).

In the study, different clinical markers were analyzed and the association has found with BS clinical score. The BS clinical score is a computed tomography, which measures pulmonary involvement, therapeutic effects and selection of patients for transplantation, which detects anatomical changes of the lung parenchyma [29,38,39]. The BS has low variation between examiners, good reproducibility, high sensitivity and specificity, and high correlation with pulmonary function test [29]. Values obtained in the BS can predict severity associated with deterioration of the structure of the lung parenchyma, which later in clinical evolution can be observed by other variables such as BMI and lung function.

The most important cause of mortality and morbidity is the increase of oxidizing reactions; in this way, understand the mechanisms associated with this process is important to the clinical dynamics of the disease and to comprehend the mechanisms by which the disease shows a variability in its severity among patients with similar *CFTR* genotype. The association of clinical, laboratory, and genetic markers, such as modifier genes, with CF’s severity can provide better understanding of clinical variability finding among these patients being important in the clinical prognosis. In addition, further studies should be developed to investigate the possibility to use products related to GSH pathway as a possible target of new drugs to CF and also to understand the oxidative process which takes place in the lungs of these patients.

## Conclusion

The described results with the support of the MDR analysis tool demonstrated a genetic interaction of CF severity with the BS, and gene polymorphisms related to GSH pathway, showing that the BS is an important marker of CF severity, since it indicates early pulmonary disease, and contribute to determine patients of a risk group without the search of other clinical and laboratory markers. In this context, polymorphisms in genes associated with metabolism of GSH act on the CF’s severity.

## Abbreviations

ACE: Angiotensin-converting enzyme; ADRB2: Beta-2-adrenergic receptor; BMI: Body mass index; BS: Bhalla score; CD14: Cluster of differentiation 14; CF: Cystic fibrosis; CFTR: Cystic fibrosis transmembrane regulator; CYP1A1: Cytochrome P450, family 1, subfamily A, polypeptide 1; DNA: Deoxyribonucleic acid; FEF25-75%: Forced expiratory flow between 25% and 75% of FVC; FEV1: Forced expiratory volume; FVC: Forced vital capacity; GCLC: Glutathione cysteine ligase, subunit catalytic; GSH: Glutathione; GSTM1: Glutathione S-transferase Mu 1; GSTP1: Glutathione S-transferase Pi 1; GSTT1: Glutathione S-transferase Theta 1; HWE: Hardy-Weinberg equilibrium; MAF: Minor allele frequency; MBL2: Mannose-binding Lectin-2; MDR: Multifactor dimensionality reduction; MLPA: Multiplex ligation-dependent probe amplification; OEGE: Online encyclopedia for genetic epidemiology; PCR: Polymerase chain reaction; RFLP: Restriction fragment length polymorphisms; TGF-β1: Transforming growth factor Beta-1; WHO: World Health Organization.

## Competing interests

Authors declare that they have no competing interests.

## Authors’ contributions

FALM: made substantial contributions to conception and design, acquisition of data, analysis and interpretation data included in this study; involved in drafting the manuscript and revising it for critically important intellectual content. CSB: carried out the molecular genetic studies and drafted the manuscript. AFR: has been involved in drafting the manuscript and revising it critically for important intellectual content. RS: carried out analyses and data interpretation, as well as has been involved in manuscript revising. JDR: made substantial contributions to conception and design, acquisition of data, and data interpretation included in this study; involved in drafting the manuscript and revising it for critically important intellectual content. All authors read and approved the final manuscript.

## Pre-publication history

The pre-publication history for this paper can be accessed here:

http://www.biomedcentral.com/1471-2350/14/60/prepub

## Supplementary Material

Additional file 1: Table S4 GCLC-129C>T polymorphism in *GCLC* gene in association with clinical variables in cystic fibrosis patients distributed by *CFTR* mutation. Click here for file

Additional file 2: Table S5 GCLC-3506A>G polymorphism in *GCLC* gene in association with clinical variables in cystic fibrosis patients distributed by *CFTR* mutation. Click here for file

Additional file 3: Table S6 The *GSTM1* gene deletion polymorphism in association with clinical variables in cystic fibrosis patients distributed by *CFTR* mutation. Click here for file

Additional file 4: Table S7 The *GSTT1* gene deletion polymorphism in association with clinical variables in cystic fibrosis patients distributed by *CFTR* mutation. Click here for file

Additional file 5: Table S8 The GSTP1+313A>G polymorphism in *GSTP1* gene in association with clinical variables in cystic fibrosis patients distributed by *CFTR* mutation. Click here for file
