# Supplementary material for: Genetic interaction of GSH metabolic pathway genes in cystic fibrosis
Source: BMC Med Genet. 2013 Jun 10;14:60. doi: 10.1186/1471-2350-14-60 (PMC3685592; doi:10.1186/1471-2350-14-60)
Supplement: Additional file 1: Table S4 — GCLC-129C>T polymorphism in GCLC gene in association with clinical variables in cystic fibrosis patients distributed by CFTR mutation. [file 1471-2350-14-60-S1.docx]

| **Table 4.** GCLC-129C>T polymorphism in *GCLC* gene in association with clinical variables in cystic fibrosis patients distributed by *CFTR* mutation | | | | | | | | |
| --- | --- | --- | --- | --- | --- | --- | --- | --- |
| Variables | Without taking *CFTR* mutation into account | | No *CFTR* mutations identified | | One *CFTR* identified mutation | | Two *CFTR* identified mutations | |
|  | p-value | p-corrected | p-value | p-corrected | p-value | p-corrected | p-value | p-corrected |
| Sex^1^ | 0.577 | 1 | 1 | 1 | 0.024 | 0.48 | 0.418 | 1 |
| Age^1^ | 0.348 | 1 | 1 | 1 | 1 | 1 | 0.249 | 1 |
| Onset of symptoms^1^ | 1 | 1 | 0.165 | 1 | 1 | 1 | 0.392 | 1 |
| Onset of pulmonary disease^1^ | 0.162 | 1 | 1 | 1 | 0.409 | 1 | 0.168 | 1 |
| Onset of digestive disease^1^ | 1 | 1 | 0.142 | 1 | 0.710 | 1 | 0.583 | 1 |
| Diagnosis^1^ | 1 | 1 | 1 | 1 | 0.715 | 1 | 0.764 | 1 |
| BMI^1^ | 1 | 1 | 0.414 | 1 | 0.331 | 1 | 1 | 1 |
| Bhalla score^2^ | 0.626 | 1 | 0.47 | 1 | 0.851 | 1 | 0.834 | 1 |
| Kanga score^2^ | 0.277 | 1 | 0.45 | 1 | 0.687 | 1 | 0.192 | 1 |
| Shwachman-Kulczycki score^2^ | 0.917 | 1 | 0.532 | 1 | 0.405 | 1 | 0.767 | 1 |
| Nasal polyposis^1^ | 0.811 | 1 | 0.66 | 1 | 0.332 | 1 | 0.066 | 1 |
| Diabetes melittus^1^ | 0.811 | 1 | 1 | 1 | 1 | 1 | 0.505 | 1 |
| Osteoporosis^1^ | 0.306 | 1 | 1 | 1 | 0.353 | 1 | 0.238 | 1 |
| Meconium ileous | 0.792 | 1 | 1 | 1 | 0.651 | 1 | 0.727 | 1 |
| Insufficiency pancreatic^1^ | 0.063 | 1 | 0.267 | 1 | 0.328 | 1 | 1 | 1 |
| SpO2^2^ | 0.384 | 1 | 0.296 | 1 | 0.124 | 1 | 0.597 | 1 |
| FVC(%)^2^ | 0.822 | 1 | 0.828 | 1 | 0.922 | 1 | 0.597 | 1 |
| FEV_1_(%)^2^ | 0.598 | 1 | 0.310 | 1 | 0.983 | 1 | 0.820 | 1 |
| FEV_1_/FVC^2^ | 1 | 1 | 0.109 | 1 | 0.873 | 1 | 0.170 | 1 |
| FEF_25-75_%^2^ | 0.448 | 1 | 0.044 | 0.88 | 0.982 | 1 | 0.537 | 1 |
| 1st *P. aeruginosa^1^* | 1 | 1 | 1 | 1 | 0.695 | 1 | 1 | 1 |
| *P. aeruginosa* mucoid^1^ | 0.133 | 1 | 1 | 1 | 0.011 | 0.22 | 1 | 1 |
| *P. aeruginosa* no mucoid^1^ | 1 | 1 | 1 | 1 | 0.266 | 1 | 0.391 | 1 |
| *A. xylosoxidans^1^* | 0.534 | 1 | 1 | 1 | 1 | 1 | 1 | 1 |
| *S. aureus^1^* | 0.261 | 1 | 0.093 | 1 | 1 | 1 | 1 | 1 |
| *B. cepacia^1^* | 1 | 1 | 1 | 1 | 1 | 1 | 1 | 1 |

*CFTR* – Cystic Fibrosis Transmembrane Regulator. *GCLC* - Glutamate cysteine ligase catalytic subunit. BMI – Body Mass Index. SpO2 = Hemoglobin oxygen saturation in the blood. FVC - Forced vital capacity. FEV_1_ - Forced expiratory volume in the first second. FEF - Forced expiratory flow between 25 and 75% of vital capacity. % - percentage. Values below 0.05 to *p* denote clinical association (bold). 1. Categorical variables – Fisher test was used. 2. Numerical variables – Student T test was used.
