# Supplementary material for: Genetic interaction of GSH metabolic pathway genes in cystic fibrosis
Source: BMC Med Genet. 2013 Jun 10;14:60. doi: 10.1186/1471-2350-14-60 (PMC3685592; doi:10.1186/1471-2350-14-60)
Supplement: Additional file 2: Table S5 — GCLC-3506A>G polymorphism in GCLC gene in association with clinical variables in cystic fibrosis patients distributed by CFTR mutation. [file 1471-2350-14-60-S2.docx]

| **Table 5.** GCLC-3506A>G polymorphism in *GCLC* gene in association with clinical variables in cystic fibrosis patients distributed by *CFTR* mutation. | | | | | | | | |
| --- | --- | --- | --- | --- | --- | --- | --- | --- |
| Variables | Without taking *CFTR* mutation into account | | No *CFTR* mutations identified | | One *CFTR* identified mutation | | Two *CFTR* identified mutations | |
|  | p-value | p-corrected | p-value | p-corrected | p-value | p-corrected | p-value | p-corrected |
| Sex^1^ | 0.753 | 1 | 0.532 | 1 | 0.149 | 1 | 0.824 | 1 |
| Age^1^ | 0.057 | 1 | 0.710 | 1 | 0.541 | 1 | 0.339 | 1 |
| Onset of symptoms^1^ | 1 | 1 | 1 | 1 | 0.731 | 1 | 0.812 | 1 |
| Onset of pulmonary disease^1^ | 0.507 | 1 | 1 | 1 | 0.727 | 1 | 0.816 | 1 |
| Onset of digestive disease^1^ | 0.865 | 1 | 0.646 | 1 | 1 | 1 | 1 | 1 |
| Diagnosis^1^ | 0.335 | 1 | 0.419 | 1 | 0.330 | 1 | 1 | 1 |
| BMI^1^ | 1 | 1 | 1 | 1 | 0.704 | 1 | 0.785 | 1 |
| Bhalla score^2^ | 0.35 | 1 | 0.830 | 1 | 0.169 | 1 | 0.495 | 1 |
| Kanga score^2^ | 0.011 | 0.22 | 0.734 | 1 | 0.067 | 1 | 0.027 | 0.54 |
| Shwachman-Kulczycki score^2^ | 0.091 | 1 | 0.725 | 1 | 0.034 | 0.68 | 0.159 | 1 |
| Nasal polyposis^1^ | 0.688 | 1 | 0.251 | 1 | 0.692 | 1 | 0.083 | 1 |
| Diabetes melittus^1^ | 0.688 | 1 | 1 | 1 | 0.419 | 1 | 1 | 1 |
| Osteoporosis^1^ | 0.133 | 1 | 1 | 1 | 0.25 | 1 | 0.335 |  |
| Meconium ileous | 1 | 1 | 1 | 1 | 1 | 1 | 1 | 1 |
| Insufficiency pancreatic^1^ | 0.698 | 1 | 0.180 | 1 | 0.376 | 1 | 1 | 1 |
| SpO2^2^ | 0.033 | 0.66 | 0.234 | 1 | 0.548 | 1 | 0.149 | 1 |
| FVC(%)^2^ | 0.412 | 1 | 0.944 | 1 | 0.036 | 0.72 | 0.955 | 1 |
| FEV_1_(%)^2^ | 0.166 | 1 | 0.877 | 1 | 0.030 | 0.60 | 0.577 | 1 |
| FEV_1_/FVC^2^ | 0.054 | 1 | 0.912 | 1 | 0.050 | 1 | 0.111 |  |
| FEF_25-75_%^2^ | 0.061 | 1 | 0.934 | 1 | 0.029 | 0.58 | 0.272 | 1 |
| 1st *P. aeruginosa^1^* | 0.350 | 1 | 0.453 | 1 | 0.716 | 1 | 0.799 | 1 |
| *P. aeruginosa* mucoid^1^ | 0.152 | 1 | 1 | 1 | 0.064 | 1 | 0.371 | 1 |
| *P. aeruginosa* no mucoid^1^ | 0.057 | 1 | 1 | 1 | 0.003 | 0.06 | 0.351 | 1 |
| *A. xylosoxidans^1^* | 0.187 | 1 | 0.066 |  | 0.565 | 1 | 0.343 | 1 |
| *S. aureus^1^* | 0.849 | 1 | 0.708 | 1 | 0.376 | 1 | 0.394 | 1 |
| *B. cepacia^1^* | 0.246 | 1 | 1 | 1 | 0.471 | 1 | 0.394 | 1 |

*CFTR* – Cystic Fibrosis Transmembrane Regulator. *GCLC* - Glutamate cysteine ligase catalytic subunit. BMI – Body Mass Index. SpO2 = Hemoglobin oxygen saturation in the blood. FVC - Forced vital capacity. FEV_1_ - Forced expiratory volume in the first second. FEF - Forced expiratory flow between 25 and 75% of vital capacity. % - percentage. Values below 0.05 to *p* denote clinical association (bold). 1. Categorical variables – Fisher test was used. 2. Numerical variables – Student T test was used.
