# Supplementary material for: Genetic interaction of GSH metabolic pathway genes in cystic fibrosis
Source: BMC Med Genet. 2013 Jun 10;14:60. doi: 10.1186/1471-2350-14-60 (PMC3685592; doi:10.1186/1471-2350-14-60)
Supplement: Additional file 3: Table S6 — The GSTM1 gene deletion polymorphism in association with clinical variables in cystic fibrosis patients distributed by CFTR mutation. [file 1471-2350-14-60-S3.docx]

| **Table 6.** The *GSTM1* gene deletion polymorphism in association with clinical variables in cystic fibrosis patients distributed by *CFTR* mutation. | | | | | | | | |
| --- | --- | --- | --- | --- | --- | --- | --- | --- |
| Variables | Without taking *CFTR* mutation into account | | No *CFTR* mutations identified | | One *CFTR* identified mutation | | Two *CFTR* identified mutations | |
|  | p-value | p-corrected | p-value | p-corrected | p-value | p-corrected | p-value | p-corrected |
| Sex^1^ | 0.171 | 1 | 0.764 | 1 | 1 | 1 | 0.110 | 1 |
| Age^1^ | 1 | 1 | 0.498 | 1 | 1 | 1 | 1 | 1 |
| Onset of symptoms^1^ | 0.268 | 1 | 0.459 | 1 | 1 | 1 | 0.629 | 1 |
| Onset of pulmonary disease^1^ | 0.424 | 1 | 0.068 | 1 | 1 | 1 | 0.639 | 1 |
| Onset of digestive disease^1^ | 0.409 | 1 | 0.665 | 1 | 1 | 1 | 0.635 | 1 |
| Diagnosis^1^ | 1 | 1 | 0.059 | 1 | 0.149 | 1 | 1 |  |
| BMI^1^ | 0.462 | 1 | 0.503 | 1 | 0.725 | 1 | 0.169 | 1 |
| Bhalla score^2^ | 0.86 | 1 | 0.11 | 1 | 0.050 | 1 | 0.879 | 1 |
| Kanga score^2^ | 0.982 | 1 | 0.693 | 1 | 0.822 | 1 | 0.784 | 1 |
| Shwachman-Kulczycki score^2^ | 0.501 | 1 | 0.449 | 1 | 0.123 | 1 | 0.568 | 1 |
| Nasal polyposis^1^ | 0.331 | 1 | 0.136 | 1 | 1 | 1 | 0.765 | 1 |
| Diabetes melittus^1^ | 0.560 | 1 | 1 | 1 | 0.703 | 1 | 0.169 | 1 |
| Osteoporosis^1^ | 0.217 | 1 | 0.435 | 1 | 0.173 |  | 1 | 1 |
| Meconium ileous | 1 | 1 | 1 | 1 | 0.726 | 1 | 0.776 | 1 |
| Insufficiency pancreatic^1^ | 1 | 1 | 0.765 | 1 | 1 | 1 | 1 | 1 |
| SpO2^2^ | 0.187 | 1 | 0.012 | 0.24 | 0.780 | 1 | 0.645 | 1 |
| FVC(%)^2^ | 0.990 | 1 | 0.741 | 1 | 0.538 | 1 | 0.967 | 1 |
| FEV_1_(%)^2^ | 0.827 | 1 | 0.623 | 1 | 0.786 | 1 | 0.943 | 1 |
| FEV_1_/FVC^2^ | 0.915 | 1 | 0.749 | 1 | 0.918 | 1 | 0.597 | 1 |
| FEF_25-75_%^2^ | 0.853 | 1 | 0.718 | 1 | 0.819 | 1 | 0.847 | 1 |
| 1st *P. aeruginosa^1^* | 0.724 | 1 | 1 | 1 | 0.056 | 1 | 0.312 | 1 |
| *P. aeruginosa* mucoid^1^ | 0.092 | 1 | 0.729 | 1 | 1 | 1 | 0.107 | 1 |
| *P. aeruginosa* no mucoid^1^ | 0.879 | 1 | 0.754 | 1 | 0.776 | 1 | 0.629 | 1 |
| *A. xylosoxidans^1^* | 0.619 | 1 | 0.537 | 1 | 0.35 | 1 | 0.52 | 1 |
| *S. aureus^1^* | 0.362 | 1 | 0.175 | 1 | 1 | 1 | 0.776 | 1 |
| *B. cepacia^1^* | 0.371 | 1 | 0.116 | 1 | 0.703 | 1 | 1 | 1 |

*CFTR* – Cystic Fibrosis Transmembrane Regulator. *GSTM1* - glutathione S-transferase mu 1. BMI – Body Mass Index. SpO2 = Hemoglobin oxygen saturation in the blood. FVC - Forced vital capacity. FEV_1_ - Forced expiratory volume in the first second. FEF - Forced expiratory flow between 25 and 75% of vital capacity. % - percentage. Values below 0.05 to *p* denote clinical association (bold). 1. Categorical variables – Fisher test was used. 2. Numerical variables – Student T test was used.
