# Supplementary material for: Genetic interaction of GSH metabolic pathway genes in cystic fibrosis
Source: BMC Med Genet. 2013 Jun 10;14:60. doi: 10.1186/1471-2350-14-60 (PMC3685592; doi:10.1186/1471-2350-14-60)
Supplement: Additional file 4: Table S7 — The GSTT1 gene deletion polymorphism in association with clinical variables in cystic fibrosis patients distributed by CFTR mutation. [file 1471-2350-14-60-S4.docx]

| **Table 7.** The *GSTT1* gene deletion polymorphism in association with clinical variables in cystic fibrosis patients distributed by *CFTR* mutation. | | | | | | | | |
| --- | --- | --- | --- | --- | --- | --- | --- | --- |
| Variables | Without taking *CFTR* mutation into account | | No *CFTR* mutations identified | | One *CFTR* identified mutation | | Two *CFTR* identified mutations | |
|  | p-value | p-corrected | p-value | p-corrected | p-value | p-corrected | p-value | p-corrected |
| Sex^1^ | 0.211 | 1 | 0.778 | 1 | 1 | 1 | 0.081 | 1 |
| Age^1^ | 0.043 | 0.86 | 0.750 | 1 | 0.083 | 1 | 0.795 | 1 |
| Onset of symptoms^1^ | 1 | 1 | 0.305 | 1 | 0.202 | 1 | 0.600 | 1 |
| Onset of pulmonary disease^1^ | 0.620 | 1 | 0.721 | 1 | 0.521 | 1 | 0.796 | 1 |
| Onset of digestive disease^1^ | 0.863 | 1 | 0.390 | 1 | 0.344 | 1 | 1 | 1 |
| Diagnosis^1^ | 0.745 | 1 | 1 | 1 | 0.068 | 1 | 0.779 | 1 |
| BMI^1^ | 0.447 | 1 | 0.747 | 1 | 0.295 | 1 | 0.537 | 1 |
| Bhalla score^2^ | 0.485 | 1 | 0.824 | 1 | 0.322 | 1 | 0.185 | 1 |
| Kanga score^2^ | 0.737 | 1 | 0.743 | 1 | 0.953 | 1 | 0.767 | 1 |
| Shwachman-Kulczycki score^2^ | 0.734 | 1 | 0.984 | 1 | 0.653 | 1 | 0.393 | 1 |
| Nasal polyposis^1^ | 0.313 | 1 | 1 | 1 | 0.062 | 1 | 1 | 1 |
| Diabetes melittus^1^ | 0.158 | 1 | 0.115 | 1 | 0.450 | 1 | 0.764 | 1 |
| Osteoporosis^1^ | 1 | 1 | 1 | 1 | 1 | 1 | 0.718 | 1 |
| Meconium ileous | 0.276 | 1 | 0.077 | 1 | 1 | 1 | 0.335 | 1 |
| Insufficiency pancreatic^1^ | 0.847 | 1 | 0.561 | 1 | 1 | 1 | 0.557 | 1 |
| SaO2^2^ | 0.988 | 1 | 0.740 | 1 | 0.595 | 1 | 0.703 | 1 |
| FVC(%)^2^ | 0.268 | 1 | 0.086 | 1 | 0.464 | 1 | 0.623 | 1 |
| FEV_1_(%)^2^ | 0.310 | 1 | 0.167 | 1 | 0.564 | 1 | 0.636 | 1 |
| FEV_1_/FVC^2^ | 0.404 | 1 | 0.288 | 1 | 0.692 | 1 | 0.424 | 1 |
| FEF_25-75_%^2^ | 0.687 | 1 | 0.390 | 1 | 0.686 | 1 | 0.959 | 1 |
| 1st *P. aeruginosa^1^* | 0.472 | 1 | 1 | 1 | 0.320 | 1 | 0.085 | 1 |
| *P. aeruginosa* mucoid^1^ | 0.433 | 1 | 0.747 | 1 | 0.393 | 1 | 1 | 1 |
| *P. aeruginosa* no mucoid^1^ | 0.876 | 1 | 0.0025 | 0.05 | 0.133 | 1 | 1 | 1 |
| *A. xylosoxidans^1^* | 0.437 | 1 | 1 | 1 | 0.623 | 1 | 0.295 | 1 |
| *S. aureus^1^* | 0.705 | 1 | 1 | 1 | 0.325 | 1 | 1 | 1 |
| *B. cepacia^1^* | 1 | 1 | 1 | 1 | 0.699 | 1 | 0.215 | 1 |

*CFTR* – Cystic Fibrosis Transmembrane Regulator. *GSTT1* - glutathione S-transferase tetha 1. BMI – Body Mass Index. SpO2 = Hemoglobin oxygen saturation in the blood. FVC - Forced vital capacity. FEV_1_ - Forced expiratory volume in the first second. FEF - Forced expiratory flow between 25 and 75% of vital capacity. % - percentage. Values below 0.05 to *p* denote clinical association (bold). 1. Categorical variables – Fisher test was used. 2. Numerical variables – Student T test was used.
