# Supplementary material for: Genetic interaction of GSH metabolic pathway genes in cystic fibrosis
Source: BMC Med Genet. 2013 Jun 10;14:60. doi: 10.1186/1471-2350-14-60 (PMC3685592; doi:10.1186/1471-2350-14-60)
Supplement: Additional file 5: Table S8 — The GSTP1+313A>G polymorphism in GSTP1 gene in association with clinical variables in cystic fibrosis patients distributed by CFTR mutation. [file 1471-2350-14-60-S5.docx]

| **Table 8.** The GSTP1+313A>G polymorphism in *GSTP1* gene in association with clinical variables in cystic fibrosis patients distributed by *CFTR* mutation. | | | | | | | | |
| --- | --- | --- | --- | --- | --- | --- | --- | --- |
| Variables | Without taking *CFTR* mutation into account | | No *CFTR* mutations identified | | One *CFTR* identified mutation | | Two *CFTR* identified mutations | |
|  | p-value | p-corrected | p-value | p-corrected | p-value | p-corrected | p-value | p-corrected |
| Sex^1^ | 0.550 | 1 | 0.396 | 1 | 0.267 | 1 | 0.184 | 1 |
| Age^1^ | 0.011 | 0.22 | 0.750 | 1 | 0.051 | 1 | 0.058 | 1 |
| Onset of symptoms^1^ | 0.876 | 1 | 0.473 | 1 | 1 | 1 | 1 | 1 |
| Onset of pulmonary disease^1^ | 0.754 | 1 | 0.729 | 1 | 0.757 | 1 | 1 | 1 |
| Onset of digestive disease^1^ | 0.516 | 1 | 1 | 1 | 0.761 | 1 | 1 | 1 |
| Diagnosis^1^ | 0.644 | 1 | 0.694 | 1 | 0.561 | 1 | 0.441 | 1 |
| BMI^1^ | 0.856 | 1 | 0.331 | 1 | 1 | 1 | 1 | 1 |
| Bhalla score^2^ | 0.098 | 1 | 0.187 | 1 | 0.491 | 1 | 0.392 | 1 |
| Kanga score^2^ | 0.716 | 1 | 0.867 | 1 | 0.407 | 1 | 0.300 | 1 |
| Shwachman-Kulczycki score^2^ | 0.554 | 1 | 0.984 | 1 | 0.73 | 1 | 0.170 | 1 |
| Nasal polyposis^1^ | 0.848 | 1 | 0.306 | 1 | 1 | 1 | 0.562 | 1 |
| Diabetes melittus^1^ | 0.336 | 1 | 1 | 1 | 0.703 | 1 | 0.582 | 1 |
| Osteoporosis^1^ | 0.159 | 1 | 0.715 | 1 | 1 | 1 | 0.009 | 0.18 |
| Meconium ileous | 0.403 | 1 | 1 | 1 | 1 | 1 | 0.161 | 1 |
| Insufficiency pancreatic^1^ | 0.581 | 1 | 0.393 | 1 | 0.703 | 1 | 0.578 | 1 |
| SpO2^2^ | 0.967 | 1 | 0.839 | 1 | 0.156 | 1 | 0.346 | 1 |
| FVC(%)^2^ | 0.441 | 1 | 0.407 | 1 | 0.849 | 1 | 0.626 | 1 |
| FEV_1_(%)^2^ | 0.338 | 1 | 0.467 | 1 | 0.907 | 1 | 0.451 | 1 |
| FEV_1_/FVC^2^ | 0.295 | 1 | 0.265 | 1 | 0.575 | 1 | 0.439 | 1 |
| FEF_25-75_%^2^ | 0.146 | 1 | 0.498 | 1 | 0.505 | 1 | 0.291 | 1 |
| 1st *P. aeruginosa^1^* | 0.035 | 1 | 1 | 1 | 0.056 | 1 | 0.203 | 1 |
| *P. aeruginosa* mucoid^1^ | 0.289 | 1 | 0.331 | 1 | 0.782 | 1 | 0.653 | 1 |
| *P. aeruginosa* no mucoid^1^ | 1 | 1 | 0.548 | 1 | 0.776 | 1 | 0.482 | 1 |
| *A. xylosoxidans^1^* | 0.806 | 1 | 0.196 | 1 | 0.350 | 1 | 0.755 | 1 |
| *S. aureus^1^* | 0.721 | 1 | 0.507 | 1 | 0.743 | 1 | 0.565 | 1 |
| *B. cepacia^1^* | 0.667 | 1 | 0.196 | 1 | 0.703 | 1 | 0.404 | 1 |

*CFTR* – Cystic Fibrosis Transmembrane Regulator. *GSTP1* - glutathione S-transferase pi 1. BMI – Body Mass Index. SpO2 = Hemoglobin oxygen saturation in the blood. FVC - Forced vital capacity. FEV_1_ - Forced expiratory volume in the first second. FEF - Forced expiratory flow between 25 and 75% of vital capacity. % - percentage. Values below 0.05 to *p* denote clinical association (bold). 1. Categorical variables – Fisher test was used. 2. Numerical variables – Student T test was used.
